# SOS Response Induces Persistence to Fluoroquinolones in *Escherichia coli*

**DOI:** 10.1371/journal.pgen.1000760

**Published:** 2009-12-11

**Authors:** Tobias Dörr, Kim Lewis, Marin Vulić

**Affiliations:** Antimicrobial Discovery Center, Department of Biology, Northeastern University, Boston, Massachusetts, United States of America; Baylor College of Medicine, United States of America

## Abstract

Bacteria can survive antibiotic treatment without acquiring heritable antibiotic resistance. We investigated persistence to the fluoroquinolone ciprofloxacin in *Escherichia coli*. Our data show that a majority of persisters to ciprofloxacin were formed upon exposure to the antibiotic, in a manner dependent on the SOS gene network. These findings reveal an active and inducible mechanism of persister formation mediated by the SOS response, challenging the prevailing view that persisters are pre-existing and formed purely by stochastic means. SOS-induced persistence is a novel mechanism by which cells can counteract DNA damage and promote survival to fluoroquinolones. This unique survival mechanism may be an important factor influencing the outcome of antibiotic therapy *in vivo*.

## Author Summary

The frequent failure of antibiotic treatments is an acute public health problem. Bacteria can escape the lethal action of antibiotics by a mutation in the cell's DNA, leading to antibiotic resistance. Alternatively, they can enter a physiological state in which the antibiotics do not affect them. This phenomenon, referred to as persistence, is different from resistance because there is no genetic modification and because it is transient. Persisters are believed to form stochastically prior to antibiotic treatment. The presence of persister cells in bacterial biofilms contributes to the difficulty in treating biofilm-related infections. We investigated the persistence of *Escherichia coli* to one of the most widely used antibiotics, ciprofloxacin. We show that the majority of persister cells are formed in response to this antibiotic, contrary to the prevailing view of persister formation. Ciprofloxacin kills bacteria by damaging their DNA. DNA damage activates a SOS gene network, the result of which is the production of various repair proteins. We uncovered a novel part of this network that leads to the formation of tolerant persister cells. The induced tolerance as a side effect of antibiotic treatment is an effective bacterial survival strategy and is likely to contribute to recalcitrance of infections.

## Introduction

Persistence is the ability of a subpopulation of susceptible bacteria to survive lethal doses of antibiotics. It is a transient and non-hereditary phenotype unlike resistance, which is due to genetic modification. The transient nature of persistence makes it inherently difficult to study therefore the underlying molecular mechanisms are still poorly understood.

Persisters are thought to be slow growing, non-growing or dormant cells, which escape the lethal action of antibiotics because their drug targets are inactivated due to the physiological state. In an *Escherichia coli* high-persistence mutant, persisters to ampicillin were shown to be non-growing prior to the addition of the antibiotic [1]. In addition, a fraction of non-growing cells was isolated from untreated exponentially growing *E. coli* and was shown to be enriched in persisters to ofloxacin [2]. These studies demonstrated that persisters can form independently of antibiotics. The switch from growing to non-growing state or dormancy, is thought to be a purely stochastic process [1],[3],[4].

Both genetic and phenotypic variability can have important consequences on bacterial survival of antibiotic treatment. One of the most prescribed broad spectrum antibiotics today are the fluoroquinolones (FQ), which target gyrase and topoisomerase. These essential enzymes regulate supercoiling of genomic DNA during replication and transcription [5],[6]. FQs prevent ligation reactions of gyrase and topoisomerase resulting in double-strand breaks (DSB) [7]. DSBs are potentially lethal DNA lesions that occur under physiological conditions through collapse of stalled replication forks, overlapping repair tracts, spontaneous breakage of DNA, and other mechanisms. *E. coli* efficiently repairs DSBs through a series of reactions carried out by enzymes participating in homologous recombination and replication [8]. Processing of DSBs leads to the induction of the SOS response. SOS is a complex network composed of more than 40 genes [9],[10]. Many of these genes are essential for efficient repair of various DNA lesions, including DSBs [11],[12].

Even though fluoroquinolones are potent bactericidal antibiotics they cannot sterilize a bacterial culture. The bulk of the population rapidly dies in response to fluoroquinolones but a small fraction persists. According to one model, persisters might survive if gyrase and topoisomerase are inactivated due to cellular dormancy [3]. Dormant cells might be expected to form stochastically during growth of a culture, prior to the antibiotic exposure [2],[13]–[15].

Alternatively, the persister state might be inducible in a cell subpopulation by exposure to the antibiotic, not stochastic and pre-existing. This could be because either dormancy is inducible, or persisters might be active and have more efficient drug efflux or more efficient repair of DSBs due to the stochastic overexpression of the genes involved in those pathways or due to the physiological events leading to the activation of the same pathways.

In order to distinguish between these possibilities, we measured numbers of persisters to the fluoroquinolone ciprofloxacin in various genetic backgrounds with altered capacity for SOS induction and DSB repair.

The majority of persisters were found to be formed upon exposure to the antibiotic and formation was dependent on the SOS DNA damage response. Contrary to the current view, a majority of surviving persisters to ciprofloxacin are not pre-existing, but induced by this antibiotic.

## Results

Fluoroquinolones (FQ) induce DSBs by interfering with the action of gyrase and topoisomerase [16]. The cellular response to DSBs primarily consists of induction of the SOS-regulon and ultimately in repair through recombination [17],[18].

According to the prevailing model [3], persisters are dormant and are formed stochastically prior to the addition of antibiotic. This suggests that persisters would not experience DSBs, would not induce an adaptive response to that type of lesion, and therefore would not need repair functions to survive. In order to test these predictions, we wanted to determine whether persisters experience DSBs and induce SOS.

**Figure 1:**
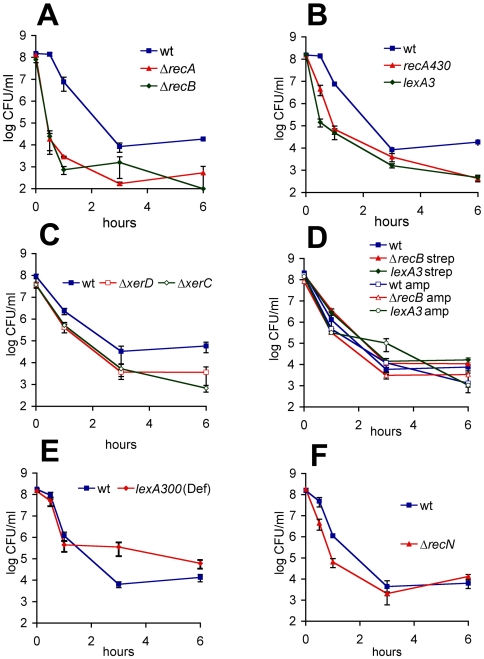
Survival of the wild type and the mutants deficient in recombination and/or SOS induction after ciprofloxacin challenge. Strains were exposed to ciprofloxacin in exponential growth phase. Viable counts were determined by plating. Graphs are representatives of at least 5 independent experiments. Error bars represent standard error.

We measured the persister levels in different genetic backgrounds diagnostic of specific molecular events linked with DSBs and SOS induction. The surviving fraction of a wild-type culture treated with ciprofloxacin produces a typical biphasic pattern (Figure 1A). This reflects the rapid killing of the bulk of the cells, and a surviving persister subpopulation.

We examined some of the well-known DNA-repair pathways in order to probe their possible role in formation of persisters. RecA and RecBC are essential for repair of DSBs in *E. coli* [19]. In strains lacking RecA and RecBC, DSBs are lethal. As expected, the bulk of cells is more rapidly killed in both *recA* and *recB* backgrounds, compared to the wild type, presumably because DSBs could not be repaired (Figure 1A). However, the persister fraction was also greatly reduced (40-fold in *recA*, 35- to 103-fold in *recB*). In *recB*, persisters were extremely rare or entirely absent after 6 hours of incubation. This shows that the persisters experience DSBs and hence depend on the repair functions.

RecA and RecB functions are essential not only for DSB-repair but for SOS induction following processing of DSBs as well [18], [20]–[22], so in order to test whether persisters induce SOS we constructed strains unable to induce SOS but proficient for homologous recombination; one carrying a non-inducible SOS-repressor (*lexA3*) [23] and the other a mutant RecA able to function as a recombinase but unable to induce cleavage of LexA (*recA430*) [24]. In both backgrounds the bulk of cells dies more rapidly than in the wild type, confirming that SOS is efficiently induced following exposure to ciprofloxacin and contributes to the survival (Figure 1B). Interestingly, the persister level is decreased 43-fold and by 6 hours it is as low as in *recA* background. This shows that the *persistence* to ciprofloxacin is largely dependent on a functional SOS response.

XerCD site-specific recombinase resolves chromosome dimers at a *dif* site [25]–[27]. Chromosome dimers are formed by an odd number of recombination events. The absence of *xerCD* function does not affect the proficiency for SOS induction, but is lethal in cells in which chromosme dimers have formed. In *xerC* and *xerD* mutants the persister level is reduced (7- and 9-fold, respectively, taking into account the 3-fold reduction in viability of *xerC* and *xerD* mutants compared to the wild-type), suggesting that most persisters have undergone at least one successful recombination event, most likely repairing a ciprofloxacin-induced DSB (Figure 1C).

Taken together these results show that the formation of the majority of persisters in the presence of ciprofloxacin is dependent on the SOS-response. They also suggest that this antibiotic-tolerant state is induced, rather than pre-existing. The formal possibility that an SOS controlled function is essential for reaching or exiting a pre-existing multidrug-tolerant state can be ruled out because tolerance to ampicillin and streptomycin were not affected in *recA*, *recB* or *lexA3* strains (Figure 1D). We cannot rule out the possibility that spontaneous SOS induction was required for creating a pre-existing ciprofloxacin-tolerant state.

A strain lacking the SOS-inducible RecN protein is also SOS proficient but partially deficient in DSB repair. *recN* mutant exhibited increased sensitivity of the bulk whereas persistence was largely unaffected (Figure 1F).

The entire population exposed to ciprofloxacin is expected to induce SOS yet only a fraction survives. SOS is a gradual response where the strength of induction reflects the extent and the persistence of the damage [28],[29]. Upon addition of ciprofloxacin the number and the chromosomal location of DSBs will vary across the population depending on the activity and position of gyrase and topoisomerase molecules in any given cell. The resulting distribution of DSBs is expected to translate into a gradient of SOS induction. Therefore, it is possible that a specific level of SOS induction is required for persistence. If this is the case, persister levels are expected to change along with ciprofloxacin concentration and the overall level of SOS induction.

**Figure 2:**
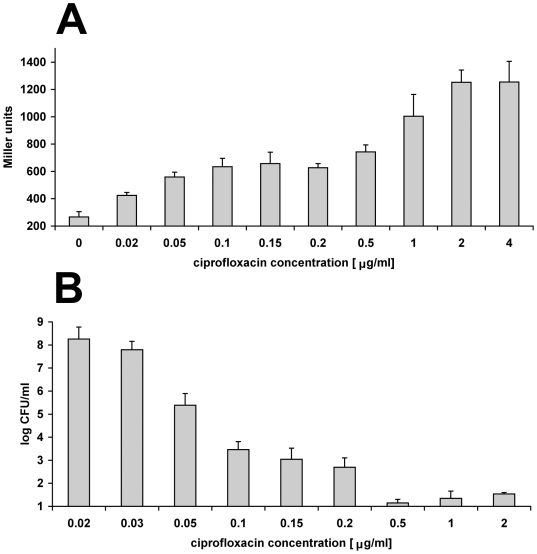
SOS induction and persister level during ciprofloxacin challenge in exponential growth phase. Graphs are averages of at least 3 independent experiments and error bars represent standard error. (A) Induction of the SOS-inducible *recA* gene expression measured by assaying the β-galactosidase activity after 15 min of ciprofloxacin challenge. (B) Persister levels after 6 hours of ciprofloxacin challenge.

We measured both the persister level and the induction of β-galactosidase under the control of an SOS-inducible *recA* promoter [30] as a population average of SOS induction for cultures exposed to increasing concentration of ciprofloxacin. Indeed, as shown in Figure 2, increased concentration of ciprofloxacin led to an increased average SOS induction (Figure 2A) and decreased persister level (Figure 2B).

A strain constitutively expressing SOS functions (*lexA300*(Def)), also led to a 20-fold increase in persister level compared to the wild type (Figure 1E).

**Figure 3:**
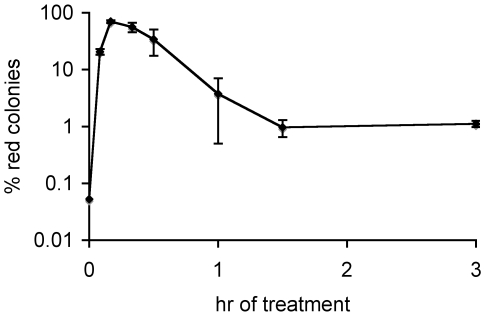
Fraction of cells undergoing strong SOS induction during ciprofloxacin challenge. Cells in exponential growth phase were exposed to ciprofloxacin. A heritable epigenetic switch based on the reciprocal repression of the phage λ *cI* and *cro* genes fused with the promotorless galactose operon allows detection of clones derived from cells that have undergone SOS induction as red *gal* ^+^ colonies on MacConkey galactose plates. Data points are average of at least 3 independent experiments. Error bars represent standard error.

In order to examine a difference in SOS induction between persisters and the bulk at the single cell level, we followed a cI-*cro gal* reporter strain after addition of ciprofloxacin [31]. In this strain the cleavage of λ repressor CI leads to a heritable genetic switch rendering a cell *gal* ^+^. *gal* ^+^ cells can be detected as red colonies on MacConkey galactose plates. Unlike LexA, which undergoes auto-cleavage early in SOS induction, CI cleavage occurs only if there is a high level of DNA damage and activated RecA [32]. Therefore, the cI-*cro* system reports conditions of only strong SOS induction. Following the addition of ciprofloxacin (>0.5 µg/ml) the proportion of cells giving rise to *gal* ^+^ colonies increases, peaking at around 20 minutes and declines thereafter (Figure 3). This timing means that the massive amount of DNA damage occurs readily leading to a strong SOS induction. Cells undergoing strong SOS induction are able to withstand and repair the damage, if the ciprofloxacin is removed by plating. However, upon extended exposure *gal* ^+^ cells become fewer (Figure 3), indicating that additional damage occurs and eventually becomes lethal. The persister subpopulation consisted almost entirely of *gal* ^−^ cells (Figure 3) showing that persisters were not SOS-induced prior to ciprofloxacin treatment (at least not highly induced) and also that they did not experience high level of DNA damage nor strong SOS induction even in the presence of the antibiotic. Because the persister level is greatly reduced in strains unable to induce SOS (*lexA3*, *recA430*, Figure 1B) we conclude that persisters undergo weak SOS induction. This is in contrast to the bulk of cells, probably because fewer DSBs occur in eventual persisters. Increased sensitivity of the bulk and minimally affected persistence in a *recN* strain also supports this conclusion (Figure 1F). SOS-inducible RecN protein promotes efficient repair of DSBs. While it is dispensable for the repair of a single break, it is essential for the repair of simultaneous multiple DSBs [33].

**Figure 4:**
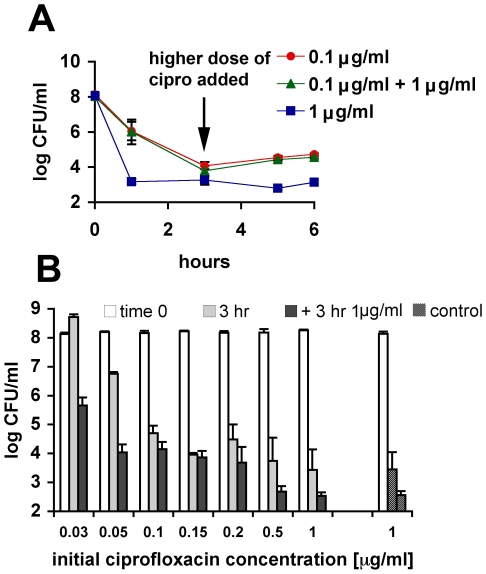
Ciprofloxacin-induced persistence. (A) Survival of wild-type cells in exponential phase under different ciprofloxacin regimes. Two cultures were treated with 0.1 µg/ml and 1 µg/ml, respectively, for 6 hours. Third culture was treated with 0.1 µg/ml for 3 hours after which 1 µg/ml was added (indicated by an arrow). The data are averages of 3 independent experiments and error bars indicate standard error. (B) Wild-type cells in exponential phase were treated for 3 hours with increasing concentrations of ciprofloxacin indicated on x-axis. After the initial treatment, an additional 1 µg/ml of ciprofloxacin was added to the cultures and incubated for another 3 hours as in (A) (ciprofloxacin MIC is 0.05 µg/ml). As a control, a parallel culture was exposed to 1 µg/ml for the duration of the experiment. Bars represent the viability at 0, 3, and 6 hr of time course equivalents shown in (A). Open bars; the initial viability count, grey bars; the viability after 3-hour incubation with ciprofloxacin concentration indicated on the x-axis. Full bars; the final viability count after additional 3-hour incubation with 1 µg/ml ciprofloxacin. Dashed bars; viability of the control culture at 3 and 6 hours. The data are averages of 3 independent experiments and error bars indicate standard error.

Next we exposed cells treated with a range of ciprofloxacin concentrations to a higher dose (1 µg/ml) of the same antibiotic (Figure 4). Control cultures were exposed to 1 µg/ml of ciprofloxacin for the duration of the experiment. The persister fraction surviving exposure to 1 µg/ml was 10- to 40-fold higher in the cultures pretreated with a low concentration of ciprofloxacin (0.05–0.2 µg/ml), compared to the control (Figure 4B). A dramatic, 1200-fold increase was found in cultures pretreated with sub-MIC (minimal inhibitory concentration) concentration of ciprofloxacin (Figure 4B; compare full bar at 0.03 µg/ml and the second dashed bar of the control). This shows that many of the persisters are formed upon ciprofloxacin treatment rather than pre-existing. If they were pre-existing, the fraction surviving the exposure to the high concentration of ciprofloxacin (1 µg/ml) would be the same regardless of the pretreatment.

**Figure 5:**
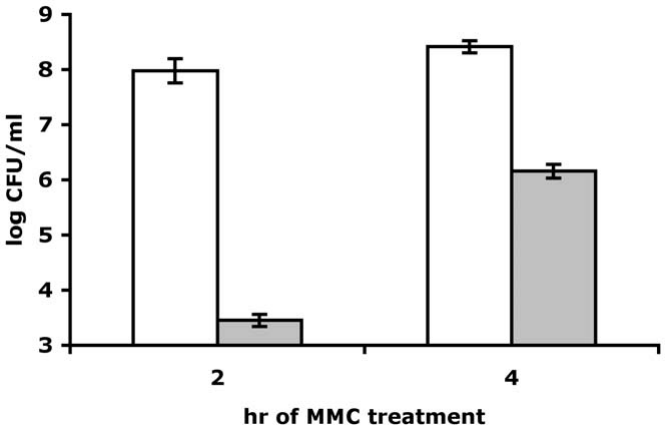
Mitomycin C-induced persistence. Wild-type cells in exponential phase were treated with mitomycin C for either 2 or 4 hours before being exposed to 0.3 µg/ml ciprofloxacin. Open bars; total viable counts, gray bars; persister fraction. The data are averages of 3 independent experiments and error bars indicate standard error.

It was important to learn whether SOS induction caused by treatments other than FQ is able to induce persistence to ciprofloxacin. In order to test this we measured persistence to ciprofloxacin in cells exposed to mitomycin C. Mitomycin C interacts with DNA by intercalation and adduct formation, resulting in inter-strand crosslinks [34]. The cellular response is a potent SOS-induction dependent on RecFOR pathway [35]. We exposed exponentially growing cells to a sub-MIC concentration of mitomycin C and compared the persister levels at two different time points during the treatment. The results in Figure 5 show a 180-fold increase in persistence to ciprofloxacin in the culture treated with mitomycin C for 4 versus 2 hours, confirming the link between SOS induction and persistence to FQs, irrespective of the nature of the SOS inducing treatment.

**Figure 6:**
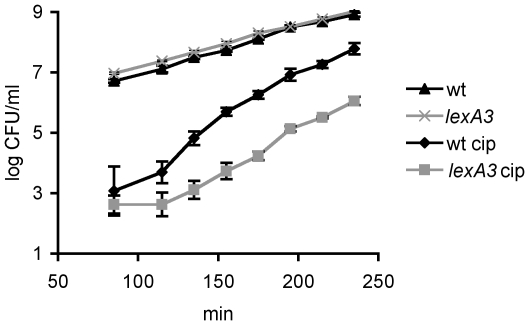
Growth phase and persister formation. Wild-type strain and strain unable to induce SOS (*lexA3*) were cultured with aeration at 37°C. A sample of each culture taken at the designated time points was treated with ciprofloxacin for 3 hrs. Cell counts before and after the antibiotic challenge were determined by plating. Data points are averages of 4 independent experiments. Error bars represent standard error.

Persister levels are very low in early exponential phase and are maximal in stationary phase [14]. We treated aliquots of growing cultures with ciprofloxacin at different time points in order to determine the persister levels between these two extremes and establish the role of growth phase in SOS-induced persistence. Figure 6 shows an exponential increase in persister levels when cell densities reach around 5×10^7^ CFU/ml in both the wild-type and the strain unable to induce SOS (*lexA3*). We conclude that the SOS-induced persisters make up the majority of persisters to ciprofloxacin regardless of the growth phase.

## Discussion

The processes leading to genetic variability in bacteria, mutagenesis and recombination, have been studied extensively [36]–[38] and their role in evolution of bacterial antibiotic resistance by generating and disseminating mutations is well established [39]–[46]. On the other hand, processes leading to phenotypic variability, which is also an important factor influencing bacterial ability to survive antibiotic treatments [47],[48] have only recently become a subject of systematic investigation. In contrast to the well-understood mechanisms of bacterial *resistance* to antibiotics, molecular mechanism(s) of *persistence* have so far remained elusive. The current model of persistence assumes that persisters are non-growing or dormant cells, formed by stochastic process(es) independently of any physiological responses normally elicited by antibiotics [1],[3],[4]. Studies involving persistence to two different classes of antibiotics, a b-lactam ampicillin [1] and a fluoroquinolone ofloxacin [2] are consistent with this model, which was therefore presumed to hold universally.

Here we show a mechanism of persister formation triggered by DNA damage inflicted by the fluoroquinolone ciprofloxacin. Formation of persisters in response to DNA damage reveals a deterministic component in this bistability phenomenon. Bistability is the stochastic production of two phenotypically distinct cell types within a clonal population of genetically identical kin cells. Bistability is observed in sporulation, competence, and motility [49]–[51]. In all cases studied, there is both a stochastic and deterministic component of bistability. Previous studies have shown that persisters can form stochastically, prior to the addition of antibiotics [1],[14]. The present findings show that persister formation can also be induced by an antibiotic, through an active process. This sheds an entirely new light on the problem of antibiotic tolerance and its role in infectious processes.

We also show that mutants defective in persistence to ciprofloxacin have normal persister levels to amipicillin and streptomycin (Figure 1D), therefore it is still possible that persistence to β-lactams is purely stochastic and not inducible. These results suggest that there are different mechanisms of persistence to different antibiotics.

Ciprofloxacin induces DSBs in cells with active gyrase and/or topoisomerase, which in turn leads to the activation of the general DNA damage stress response, the SOS gene network. Our results show that the majority of persisters to ciprofloxacin are dependent on a functional SOS response.

DSBs and other SOS-inducing lesions occur under physiological conditions so at any given time there is a fraction of a bacterial population undergoing a certain degree of SOS induction [52],[53]. However, we demonstrate that the SOS-dependent persister state is induced upon exposure to ciprofloxacin. Manipulating the extent of SOS induction by different antibiotic concentrations or by sequential exposure to a higher dose dramatically affects persister levels (Figure 2, Figure 4). This would not be the case if the persisters were pre-existing in the population. If they were, the bulk would be killed by any bactericidal concentration of antibiotic, revealing the same pre-existing persister population. In addition, increasing the basal level of expression of the SOS regulon by genetic manipulation (Figure 1E) or by induction with different treatment (Figure 5) also leads to an increase in persister level.

Essentially all actively growing cells exposed to ciprofloxacin induce SOS, but not all become persisters, suggesting that a specific level of SOS induction is required for persister formation. SOS is a gradual response and depending on the nature of the inducer, its concentration and the time of the exposure, different sets of genes are induced [10],[54],[55]. Our data indicate that persister formation requires a functional SOS response but a high level of induction is not required (Figure 1B, Figure 3). Persister formation also depends on functional DSB repair (Figure 1A) but does not need RecN (Figure 1F), a function important for the repair of multiple DSBs [33]. This implies that persisters are cells that experienced few DSBs upon ciprofloxacin addition and underwent weak SOS induction.

Consistent with this, constitutive, full expression of the SOS regulon (equivalent to high induction) does not lead to the tolerance of the entire population, but to an increased level of surviving persisters (Figure 1E). Even in a *lexA*(Def) mutant, expression levels of SOS genes appear to fall short of being truly uniform throughout the population [52],[53]. Persisters could be the cells that express a certain SOS function at a specific high or low level. Additionally, other regulatory pathways could allow a persister formation function to be expressed only in certain cells after induction. Turning on the SOS response constitutively would increase the number of cells being able to express this function.

Persister levels are known to change with the growth phase [14]. It is low in early exponential phase and attains its highest level in stationary phase. An exponential increase in persister levels begins when the cell density reaches around 5×10^7^ CFU/ml (Figure 6). The persister shoot up at similar cell density has been observed in other studies under different antibiotic and growth conditions [1],[14]. A cell density of 5×10^7^ CFU/ml coincides with the point at which the balanced growth of the culture ceases and a slowdown of growth rate is observed, even though the population as a whole still increases exponentially [56].

The extent of the DNA damage caused by ciprofloxacin would be expected to reflect the activity levels of gyrase and topoisomerase. These enzymes are active during replication and transcription [6],[57]; therefore their maximal activity would occur in rapidly growing and replicating cells and would be lowest in the non-growing state of stationary phase. Lending support to this, transcription of *gyrA* and *gyrB* coding for gyrase subunits is at the peak in the early exponential phase and the lowest in the stationary growth phase [58],[59]. It follows that ciprofloxacin would inflict maximal damage, the irreparable chromosome fragmentation, in the exponentially growing cells and fewer DSBs in the cells that slow down when the medium cannot support steady-state growth [60]. Indeed, no cells survive treatment to ofloxacin, another FQ, when the culture is kept at low density in constant exponential growth by repeated subculturing [14], in other words no persisters are formed in that growth phase. On the other hand, the surviving fraction increases dramatically between the end of true exponential growth and stationary phase (Figure 6, [14]). During that time the growth rate of the population decreases from its maximum to zero, but because not all cells stop growing at the same time the heterogeneity of growth rates across the population is expected within that time frame. Those cells lacking steady state equilibrium might be the ones which experience few DSBs, weak SOS-induction and enter the tolerant state. Consistent with this, the difference in persister level in SOS proficient and deficient strains is minimal in early exponential phase, whereas it increases after the cessation of steady state growth (Figure 6).

Conditions for unrestricted growth are rarely met in natural environments, and most bacteria are in a state of slow or no-growth [61]–[63]. However, physical and chemical agents capable of causing DNA damage are ubiquitous, therefore the SOS-induced persister state is probably quite common. Furthermore, in conditions of slow growth and frequent or lasting presence of DNA damaging agents, damage prevention would likely be advantageous over continuous active repair. The induction of the persister state in response to DNA damage seems like such a strategy - the avoidance of the damage build up as opposed to the costly repair.

SOS is induced in aging colony biofilms of *E. coli* [64] and in intracellular biofilms formed by uropathogenic *E. coli* during cystitis [65]. Biofilms are notoriously hard to eradicate even with bactericidal fluoroquinolones, and this enhanced ‘resistance’ could in fact reflect the SOS-induced tolerance.

Virtually all natural isolates of *E. coli* and many other bacteria are lysogens and many prophages are DNA-damage inducible [66]–[69]. Induction of λ prophage in *E. coli* is a late SOS function. In that light, SOS-induced tolerance could have evolved as a life-saving strategy preventing prophage induction upon DNA damage frequently encountered.

There are at least 43 genes in the *E. coli* genome negatively regulated by LexA [9],[10]. Many encode proteins participating in repair by homologous recombination and/or translesion synthesis and about one third are of unknown function. Among those are several genes encoding toxin-antitoxin modules that are attractive candidates for persistence genes, as the overexpression of some toxins has been shown to induce a dormant-like state [13],[70]. Indeed, in a parallel study we identified an SOS inducible toxin/antitoxin module, *tisAB*, as a function needed for persister formation (Dörr T., Vulić M., Lewis K., submitted). However we cannot exclude that other LexA-regulated genes also contribute to SOS-induced tolerance.

SOS has been shown to induce formation of a senescence-like state in which cells are viable but unable to form colonies [53]. Here we show SOS-dependant formation of persister cells. Both states could be formed through the common mechanism, such as expression of SOS-regulated toxins. In that case the strength of SOS induction and hence the toxin expression levels would determine which of these two states a cell reaches.

In conclusion, we have discovered an active, regulated mechanism of persister formation, which is part of the SOS response. SOS has been known to contribute to the survival of antibiotic treatments by increasing the frequency of resistant mutants through its mutagenic activities [44],[71]. Here we show a novel function of this response, the induction of a tolerant state. SOS-induced persistence having an immediate impact on bacterial survival is likely an important factor influencing the outcome of antibiotic treatment.

## Materials and Methods

### Bacterial strains

Bacterial strains are listed in Table 1. Wild-type *E. coli* K-12 MG1655 was used as the parental strain. Different alleles were moved into the parental background by P1 transduction [72]. The kanamycin resistance cassette from the alleles originated from KEIO collection [73] was cured when needed by expressing the FLP recombinase from the helper plasmid pCP20 according to the protocol in [74].

**Table 1:** Bacterial strains.

Strain	Relevant genotype	Parent strain	Reference		Source
MG1655	K-12 F^−^ λ^−^				
TD172	Δ*recA*::kan	JW2669	[73]		
TD230	Δ*recB*::kan	JW2788	[73]		
TD160	Δ*recN*::kan	JW2597	[73]		
TD222	*recA430*	GY3448	[24],[75]	*E. coli* Genetic Stock Center, Yale	
TD221	*lexA3 malE300*::Tn*10*	K996	[23]	*E. coli* Genetic Stock Center, Yale	
TD127	*lexA300*(Def) Δ*sulA*::FRT	GW8018, JW0941	[73],[76]	Walker Lab, MIT, Cambridge	
MV2033	Δ*xerD*::kan	JW2862	[73]		
MV2037	Δ*xerC*::kan	JW3784	[73]		
LLC3	(λ cI^+^ *cro* ^+^-*gal* ^−^)	MT1	[31]	Radman Lab, Necker, Paris	
MV1603	λ d(*recA*::*lacZ*) *cI*(Ind^−^) Amp^R^	AB1157 λ d(*recA*::*lacZ*) *cI*(Ind^−^) Amp^R^	[30]	Radman Lab, Necker, Paris	

### Persistence assay

Experiments were conducted at 37°C in Mueller Hinton Broth (MHB) supplemented with 10 mg/L MgSO_4_ and 20 mg/L CaCl_2_ according to NCCLS (National Committee for Clinical Laboratory Standards) guidelines for susceptibility testing and 0.1 M HEPES/KOH pH 7.2.

Persistence was measured by determining survival upon exposure to 0.1 µg/ml ciprofloxacin (unless indicated otherwise), 100 µg/ml ampicillin and 25 µg/ml streptomycin during time indicated on corresponding graph axes. All antibiotics were purchased from Sigma.

Prior to the addition of antibiotic overnight cultures were diluted 100-fold in 3 ml of fresh medium in 17- by 100-mm polypropylene tubes and incubated for 1.5 hrs with shaking, typically reaching ∼2×10^8^ CFU/ml. For determination of CFU counts, cells were washed in 1% NaCl solution, serially diluted and plated on LB (Luria-Bertani medium) agar plates supplemented with 20 mM MgSO_4_.

Persister fraction, reflected as a plateau in CFU counts, was calculated as an average of CFU counts at 3- and 6-hour time points. In *lexA3* and *recA430* strains the CFU counts stabilize later than in the wild-type and in that case the CFU counts at 6-hour time point were used as a representative of the persister fraction.

### Measurements of SOS induction

For plate assays using the CI-*cro*-*gal* construct, overnight cultures grown in LB medium at 37°C were diluted 1∶200 in 15 ml of fresh medium and incubated in 125 ml flasks for 1.75 hrs at 37°C with shaking. Ciprofloxacin was added and aliquots of the culture were taken at different time points, washed in 1% NaCl solution, serially diluted and plated on LB agar plates supplemented with 20 mM MgSO_4_ for total CFU counts and on MacConkey agar plates supplemented with 1% galactose in order to determine the fraction of *gal* ^+^ cells.

For β-galactosidase activity measurement, overnight cultures grown in supplemented MHB medium (see above) at 37°C were diluted 1∶100 in 3 ml of fresh medium in 17- by 100-mm polypropylene tubes and incubated for 1.75 hrs at 37°C with shaking. Ciprofloxacin was added and after 15 minutes an aliquot of culture was taken and *recA*::*lacZ* expression was measured as described in [72].

### Mitomycin C treatment and persistence to ciprofloxacin

Overnight cultures in supplemented MHB medium were diluted 1∶1000 in 15 ml of fresh medium and incubated in 125 ml flasks for 1 hr at 37°C with shaking after which 0.25 µg/ml of mitomycin C (Sigma) was added to the cultures. This concentration did not inhibit the growth of the culture. After 2 hrs the total CFU counts were determined by dilution and plating and an aliquot of the culture was taken out and exposed to 0.3 µg/ml of ciprofloxacin for 3 hrs. The number of survivors was determined by plating on LB agar plates supplemented with 20 mM MgSO_4_ after washing in 1% NaCl solution. The same procedure was repeated after 4 hours of exposure to mitomycin C.

### Kinetics of persister formation

Overnight cultures in MHB medium were diluted 1000-fold in 100 ml of fresh medium in 500 ml flasks and incubated at 37°C with shaking. At defined time intervals the cultures were serially diluted and plated on LB agar for determination of total CFU counts. In the same time 1 ml aliquots were transferred into 2 ml eppendorf tubes and 0.1 µg/ml ciprofloxacin was added. After 3 hrs at 37°C cells were washed with 1% NaCl solution, serially diluted and plated on LB agar plates supplemented with 20 mM MgSO_4_. The colonies were counted after 40 hours incubation at 37°C.
